# Additive Manufacturing of Geopolymers Modified with Microalgal Biomass Biofiller from Wastewater Treatment Plants

**DOI:** 10.3390/ma12071004

**Published:** 2019-03-27

**Authors:** Emanuele Agnoli, Riccardo Ciapponi, Marinella Levi, Stefano Turri

**Affiliations:** 1 Department of Chemistry, Materials and Chemical Engineering “Giulio Natta”, Politecnico di Milano, Piazza Leonardo da Vinci 32, 20133 Milano, Italy; marinella.levi@polimi.it (M.L.); stefano.turri@polimi.it (S.T.); 2 INSTM—National Interuniversity Consortium of Materials Science and Technology, Via G. Giusti 9, 50121 Firenze, Italy; riccardo.ciapponi@polimi.it

**Keywords:** additive manufacturing, 3D printing, liquid deposition modeling, geopolymers, metakaolin, biofillers, end-of-life materials, microalgae, SaltGae

## Abstract

This paper deals with the additive manufacturing of metakaolin-based geopolymers and with the use of microalgal biomass from wastewater treatment plants as biofiller in this kind of cementitious material. The study was developed following the evolution stages of the material, which was prepared and printed as a soft paste and then hardened thanks to an inorganic polymerization reaction (geopolymerization). Thus, the characterization techniques adopted encompassed rheometry, mechanical tests performed on the hardened material, scanning electron microscopy (SEM), energy-dispersive X-ray spectroscopy (EDS) and mercury intrusion porosimetry (MIP). Microalgal biomass addition, evaluated in this study at 1, 3 and 5 php with respect to the powder weight, affected both the properties of the fresh and of the hardened material. Regarding the former aspect, biomass reduced the yield stress of the pastes, improving the ease of the extrusion process, but potentially worsening the ability to build structures in height. When hardened, geopolymers containing microalgae showed mechanical properties comparable to the unfilled material and a microstructure characterized by smaller pores. Finally, a printing test was successfully performed with a larger printer to assess the feasibility of producing large-scale structures. Taking into account these results, this study demonstrates the possibility of using microalgal biomass as biofiller in geopolymers for additive manufacturing.

## 1. Introduction

The possibility to produce objects directly from a 3D digital model has made additive manufacturing (AM), also popularly known as 3D printing, attractive for a wide range of applications in many different sectors. Traditionally known as a technology suitable for making prototypes and mockups rapidly, recently AM has become interesting to obtain easily customizable, functional final product [1,2,3,4].

One of the most promising application of AM is in the construction field, which is already moving toward a digitalization of processes at the design phase, with the use of building information models (BIM), and is seeking for automation also in the construction phase [5]. AM can improve this sector, by introducing a new freedom in design, and by reducing manpower, construction time, material consumption and, consequently, costs [6,7].

3D printing of cementitious materials has been studied and, in some cases, scaled-up to obtain real buildings. AM technologies in this field can be divided in two broad categories: powder-based and extrusion-based. In techniques following the former approach, a liquid binder is jetted on a powder bed to make particles stick together, building the object layer-by-layer; high resolution and mechanical properties can be achieved, but the printing speed is low, and the dimension of printed object is limited by that of the frame containing the powder bed [8,9]. Conversely, in extrusion-based processes the cementitious material is extruded in a fresh state to build freeform structures layer-by-layer; although resolution is lower with respect to powder-based techniques, printing speed is much higher and in principle, there are no limitations on the dimensions of the structure [10,11]. Because of these aspects, in this paper an extrusion-based process, called liquid deposition modeling (LDM) [12,13], is studied.

A cementitious paste for LDM should comply mainly with two requirements: printability and buildability. From the printability point of view, the paste must be extruded easily and in a reliable way, forming continuous filaments, avoiding plugging at the nozzle and too high of a pressure build-up that may significantly affect the printing quality. Buildability, on the other hand, can be expressed as the ability of the material to sustain the loads due to the subsequent layers deposited. In addition, open time, that is the period of time in which a mortar is workable, must be taken into account to avoid the hardening of the cementitious material in the printing system [14,15].

Along with the automation of the building technologies, advancement in construction industry must pass through the evolution of building materials. Nowadays, ordinary Portland cement (OPC)-based concrete is the most widely used construction material. Current average consumption of concrete is about 1 t/year per every living human being, second only to water consumption. Due to its large demand, even small reductions of greenhouse gas emissions per ton of manufactured concrete can make a significant global impact. Since the production of 1 ton of OPC implies the emission of about 0.5 tons of carbon dioxide and an intensive use of energy, the easiest solution to reduce the environmental impact of construction industry is shifting to alternative cementitious binders [16,17]. In recent years geopolymers, also called alkali-activated cements (AAC), are emerging as promising cementitious materials to provide an environmentally friendly alternative to OPC, since the production of raw materials is less polluting and energy-intensive than OPC [16,18,19,20,21,22]. In particular, from the comparison of the life cycle assessment (LCA) studies present in literature, Habert and Oullet-Plamondon concluded that AAC can reduce global warming potential (GWP), i.e., the environmental impact category related to greenhouse gases emissions, by a factor of 4 compared to OPC [23]. Regarding other environmental impact categories, related to nitrogen cycle and biodiversity loss, benefits are less clear. Specifically, further research developments are needed to improve the sustainability of the alkaline activation process, using wastes instead of pure materials to obtain the precursors for alkaline solutions [18,23].

Typically, geopolymers are obtained by mixing an aluminosilicate source (e.g., metakaolin, fly-ash or granulated blast furnace slag) with an alkaline solution (called alkaline activator); alkaline environment is needed to dissolve aluminosilicates and to trigger a polycondensation reaction (geopolymerization). Passing through various steps, particles coagulate and condense to form a polycrystalline or amorphous solid material [24,25,26,27,28,29].

To reduce the environmental impact of concrete further, waste materials (inorganic and organic) can be adopted in cement industry as fillers and even property modifiers [19,30,31]. This paper deals with microalgal biofillers, which are wastes of innovative wastewater treatment plants based on filtration by microalgae. These plants take advantage of the naturally occurring phenomenon of eutrophication: in presence of water containing high concentrations of nutrients, the growth of algae is highly promoted. In natural environments, eutrophication can cause the death of other organisms due to the reduction of the oxygen level, but this phenomenon can be positively used in wastewater treatments to remove nutrients and heavy metals from wastewaters before their discharge [32,33,34].

This work is part of the project SaltGae, funded by EU in the framework of Horizon 2020 research and innovation program. The aim of the project is to implement and demonstrate at large scale the long-term technological and economic feasibility of an innovative, sustainable and efficient solution for the treatment of high salinity wastewater based on the use of microalgae. In addition, in order to minimize the economic and environmental impact of the treatment, SaltGae includes in its workplan the valorization of end-of-life algal biomass into different by-products for high value-added applications [35].

Therefore, the aim of this work is related to the byproducts’ valorization aspect. In particular, geopolymers for LDM technology were studied, focusing on the effect of microalgal biomass addition on the properties of fresh and hardened material.

## 2. Materials and Methods

### 2.1. Raw Materials

To produce geopolymer formulations for LDM, metakaolin, an alkaline activator composed of sodium silicate and sodium hydroxide solutions, bentonite and distilled water were used. Two microalgal biomass species (*Spirulina platensis* and *Tetraselmis suecica*) and lignin were added in different amounts to the mixtures. Lignin was used as a reference biomass, available as waste from the paper industry, to be compared with microalgal species.

The metakaolin used in this work, with the commercial name Mefisto L05, was provided by České Lupkové Závody (Pecínov, Czech Republic). The specific surface area of the powder was 12.69 m^2^/g, LOI 2.20%, d_50_ 3 µm and d_90_ 10 µm. The chemical composition guaranteed by the supplier is shown in Table 1.

A commercial solution-based alkaline activator, with a formulation based on sodium silicate and sodium hydroxide, was purchased by České Lupkové Závody (Pecínov, Czech Republic), in bundle with the metakaolin powder. Thermogravimetric analysis performed on the activator solution showed that the water content is 62 wt %.

Bentonite, which is an absorbent aluminum phyllosilicate clay consisting mostly of montmorillonite, was used in geopolymer pastes as a rheology modifier, to increase plasticity and to obtain a proper viscosity. A commercial bentonite, “Mapeproof Seal”, was purchased by Mapei (Milano, Italy).

Microalgal biomasses, *Spirulina platensis* and *Tetraselmis suecica*, were kindly provided by the SaltGae partner Archimede Ricerche (Camporosso, Italy) as freeze-dried powders. Chemical analyses on *Spirulina* and *Tetraselmis*, performed by the partner Extractis (Dury, France), are reported in Table 2.

The softwood Kraft lignin used in this work, “Indulin AT”, was provided by MeadWestvaco (Richmond, VA, USA) as a powder.

### 2.2. Formulations

Geopolymer mixtures for LDM were optimized in order to comply with the requirements of printability and buildability. These aspects strongly depend on the printing setup. Therefore, passing from an extrusion system to another (e.g., from syringe-based to screw-based extruders) or changing the scale of printing may require the adjustment of mixture proportions.

Table 3 reports the mixture compositions analyzed in this work. All formulations were based on the mixture of the metakaolin and the alkaline activator in a fixed mass ratio of 5/4. Bentonite and water were added to adjust rheological behavior. To investigate the effect of biomass addition on geopolymer properties, three different biomass contents were selected: 1, 3 and 5 per hundred parts (php) with respect to the powder weight, i.e., metakaolin and bentonite. These numbers are reported in the formulation ID, next to a letter indicating the biomass type (“S” for *Spirulina*, “T” for *Tetraselmis*, “L” for lignin). Higher quantities of biomass led to too stiff and dry mixtures, difficult or impossible to print. Formulations containing biomass were compared to the unmodified geopolymer mixture (G-0). The formulation U-S5, containing *Spirulina* by 5 php, was tuned for the scale-up printing (refer to Section 2.3).

The powders (i.e., metakaolin, bentonite and biomass) were weighed in a plastic beaker and manually mixed with a spatula for about 1 min. Then, the liquids were weighed and added in the same beaker. Liquids and powders were incorporated by mixing them manually with a spatula for about 5 min, until a uniform paste was obtained. An electric mortar mixer was used for the larger scale printing test.

### 2.3. Printing Setups and Preparation of the Specimens

**Figure 1:**
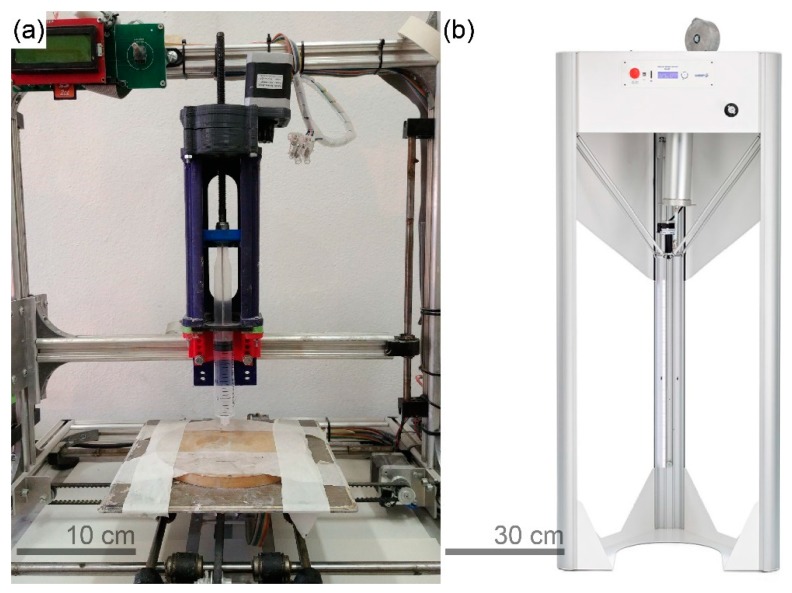
3D printing setups adopted in this work: (**a**) 3DRag modified for LDM; (**b**) Delta WASP 40100 Clay.

A desktop fused deposition modelling (FDM) 3D printer, 3DRag, produced by Futura Elettronica (Gallarate, Italy), was modified for LDM by substituting the FDM extruder with a piston-type extruder (Figure 1a). The extrusion apparatus was designed to support many commercial syringes, ranging from 10 to 100 mL capacity. A 20 mL syringe, with a nozzle diameter of 2.25 mm, was equipped to the system to print the specimens for compression tests. Samples with hollow cylindric shape (15 mm external diameter, 10.5 mm internal diameter, 28 mm height) were printed on a metallic support; six specimens were obtained for each batch. Printings were conducted in spiral mode, a printing mode that converts the subsequent layers in a continuous spiral; layer height was set at 0.6 mm and printing speed at 20 mm/s.

Samples were cured at ambient temperature for 7 or 28 days, covered under a plastic sheet. After the ambient curing, some specimens were thermally treated at 800 °C for 4 h in a muffle oven (model 10-D1418/A, produced by Controls, Liscate, Italy).

The feasibility of printing structures in a scale larger than that possible with a desktop printer was assessed by a trial scale-up printing. For this purpose, a Delta WASP 40100 Clay, produced by WASP (Massa Lombarda, Italy), was used (Figure 1b). Its larger printing volume of 40 cm × 40 cm × 100 cm, if compared with the dimension of 3DRag (20 cm × 20 cm × 20 cm), allowed larger printed structures, developed mainly in height. Unlike 3Drag, Delta WASP 40100 Clay was provided with a screw extrusion system, fed by a closed container kept under pressure, and a 4 mm-diameter nozzle. Different printing parameters were set, as well: layer height was 2 mm and printing speed 85 mm/s.

### 2.4. Characterization Techniques

#### 2.4.1. Rheological Measurements

Rheological characterization was carried out to understand the behavior of the pastes during the extrusion and how they were influenced by biomass. The measurements were performed by the stress-controlled rotational rheometer Kinexus Pro+ (Malvern Panalytical, Malvern, UK) equipped with the 20 mm-diameter plate-plate geometry. The tests were carried out according to the following procedure: after a pre-shear at rate 100 s^−1^ for 25 s and a 60 s-rest, a ramp at 125 Pa/s was applied. The pre-shear and the consequent rest were used to normalize the starting condition of the samples. For each formulation, measurements were performed on at least three different samples; if the results were repeatable, the average stress-shear rate curve was considered for data analysis purposes.

#### 2.4.2. Mechanical Characterization

Mechanical properties were assessed by compression tests done on the hardened specimens. The experiments were performed in displacement control, with the dynamometer ZwickRoell ProLine Z010 (ZwickRoell, Ulm, Germany), equipped with a 10 kN load cell and square plates. After a preload of 5 N, displacement was applied at constant rate of 1 mm/min in the same direction of the printing process. The test ended when the specimen displayed a well-defined fracture pattern. For each specimen, compressive strength was calculated as the ratio between the maximum load sustained and the original cross-sectional area. At least five printed specimens were tested for each formulation.

#### 2.4.3. Microstructural and Compositional Analyses

Microstructure morphology was investigated by scanning electron microscopy (SEM) analyses performed on geopolymer fragments belonging to fractured compression tests specimens (after 28 days of curing). The scanning electron microscope used in this work was an EVO 50 Extended Pressure, produced by ZEISS (Oberkochen, Germany). The experiments were performed in low-vacuum regime (chamber at 50 Pa); the investigated surface was obtained by N_2_-induced brittle fracture; the electron beam was produced by a LaB_6_ nanocrystal source and electrons were accelerated by a potential difference of 20 kV. The experimental apparatus was equipped with a spectroscope for energy-dispersive X-ray spectroscopy (EDS). This technique was used to perform compositional analyses in precise spots of the exposed surface.

The study of porosity was carried out by mercury intrusion porosimetry (MIP) with a MicroActive AutoPore V 9600 (Micromeritics Instrument Corp., Norcross, GA, USA). As for SEM and EDS, the analyses were made on geopolymer fragments belonging to fractured compression tests specimens.

## 3. Results and Discussion

### 3.1. Printability and Buildability

The rheological behavior of the pastes for LDM applications is of paramount importance for the quality and the structural properties of the built object. As already discussed, the most important aspects to take into account for the fresh material are printability and buildability. The former is related to the ease and the reliability of the extrusion process, while the latter refers to the ability of the fresh material to sustain the weight due to the layers deposited upon it.

These aspects can be related to the rheological properties of the material, in particular to yield stress. Perrot et al. [36] proposed a relation between buildability and the yield stress of the material deposited in the first layer, which is:

(1) $$H=\frac{\alpha }{\rho g}\tau_{y}$$

where *H* is the maximum height of the structure, $\tau_{y}$ is the yield stress, α is a geometrical parameter depending on the shape of the built structure, ρ is the density, and g is the gravity acceleration. Therefore, from this perspective, an improvement of buildability can be achieved by increasing the yield stress.

Nevertheless, since the flow begins when the yield stress is exceeded, a fluid with high yield stress may be too difficult to extrude, causing phenomena like plugging and rupture of the syringe, due the high pressure build-up. So, even though a yield stress is necessary to build a self-standing structure, it should be not higher than the maximum shear stress that the extrusion system can provide. Panda et al. [37] found an optimal range of printability for geopolymer concrete, which is in the order of 1–2 kPa for the yield stress.

**Figure 2:**
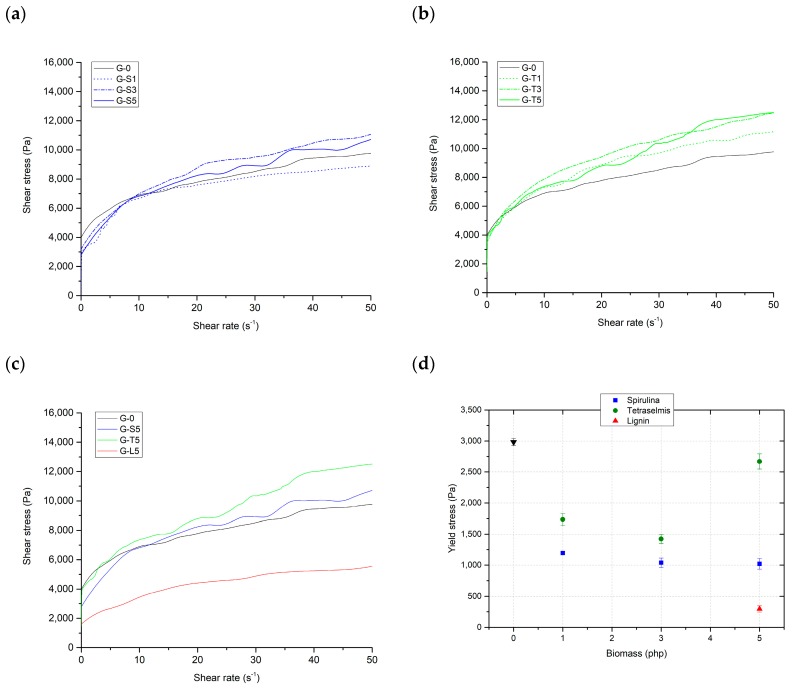
Results of the rheology tests performed on geopolymer pastes. (**a**) Flow curves of samples containing different amounts of *Spirulina platensis*; (**b**) flow curves of samples containing different amounts of *Tetraselmis suecica*; (**c**) comparison between flow curves of samples with different biomasses (*Spirulina*, *Tetraselmis* and lignin) at 5 php addition; (**d**) values of yield stress obtained by fitting the flow curves with Hershel–Bulkley’s model.

In Figure 2 the flow curves of geopolymer pastes containing different amounts of *Spirulina*, *Tetraselmis* and 5 php lignin are reported. Looking at the curves it can be noticed that the behavior of the fresh mortar is non-Newtonian (pseudo-plastic), showing yield stress, that can be found at the intercept with the ordinate axis. This kind of rheological behavior can be described by Herschel–Bulkley’s model, which can be expressed by the power-law relation:

(2) $$\tau =\tau_{y}+K{\dot{\gamma }}^{n}$$

where τ is the shear stress, $\tau_{y}$ is the yield stress, K is the consistency, $\dot{\gamma }$ is the shear rate, and n is the flow index. Flow curves were fitted according to Herschel–Bulkley’s model to find the yield stress and the parameters K and n. The results are shown in Table 4.

Fitting with Herschel-Bulkey’s model was very successful (R^2^ between 0.952 and 0.997), confirming that the pastes were pseudo-plastic fluids showing yield stress. This rheological behavior is typical of solid dispersed phases organized in continuous three-dimensional structures (coagulated structures), which are broken by the application of stresses higher than a certain threshold (the yield stress). In these systems, yield stress depends on the nature, the concentration, and the interactions between the solid particles dispersed in the liquid medium [38]. In the case of this study, the dispersing medium was constituted by the alkaline activator solution and water, while the dispersed particles were metakaolin, bentonite and biomass flocs.

Biomass deeply affects the value of the yield stress, for any species and concentration investigated. The yield stress is indeed reduced in any case, because biomass flocs, which do not interact with the dispersed aluminosilicate particles, actually hinder the geopolymer gel interactions. This causes a reduction in the resistance of the coagulated structures and, as a consequence, of yield stress. As shown graphically in Figure 2d, the decrease is monotonic in the case of *Spirulina*, but shows a minimum for *Tetraselmis*; the final increase of yield stress found at 5 php of *Tetraselmis* is probably due to the absorption of water by biomass, which rises the actual concentration of the system. In the case of lignin, which was tested only at 5 php, the reduction of yield stress is much more pronounced.

The decrease of the yield stress is beneficial for the printability, but it worsens buildability. Anyway, the values obtained with *Spirulina* and *Tetraselmis* are still acceptable, referring to the aforementioned findings of Panda et al. [37]. Conversely, lignin reduces too much the yield stress, therefore precluding the possibility to develop self-sustaining structures in height.

### 3.2. Mechanical Properties of the Hardened Material

**Figure 3:**
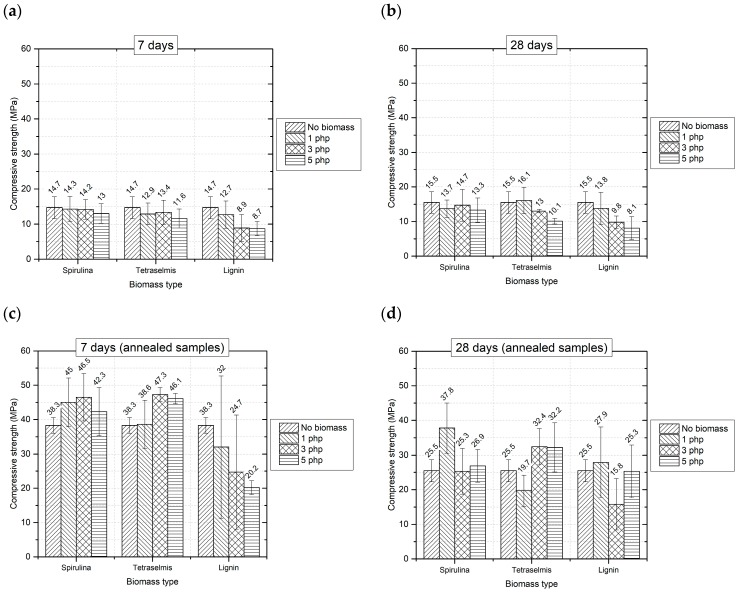
Mechanical properties of the hardened materials tested at different curing times and conditions: (**a**) after 7 days of ambient curing; (**b**) after 28 days of ambient curing; (**c**) after 7 days of ambient curing and thermal treatment; (**d**) after 28 days of ambient curing and thermal treatment.

The assessment of structural properties is crucial in sight of the application of these materials in the building and construction sector. Figure 3 reports the average values of compressive strength obtained by compression tests.

Regarding non-annealed samples, no noticeable difference exists between values of compressive strength at 7 and 28 days of ambient curing. This is not surprising, since geopolymers are known as fast-setting cementitious materials, so most of the mechanical properties may be already achieved after 7 days [39].

The addition of microalgae to the geopolymer slightly reduces mechanical properties both at 7 and at 28 days (Figure 3a,b). This reduction is negligible in any case when *Spirulina* is added, as far as the standard deviations are considered. The same consideration holds also for *Tetraselmis* up to 3 php; at 5 php compressive strength is reduced with respect to the reference formulation G-0 about 21% at 7 days and 35% at 28 days.

Annealing leads to a significant improvement of mechanical properties for all the samples tested. Indeed, at the selected temperature (800 °C), thermal treatment induces structural changes, mainly related to sintering effects, and the reaction of unreacted particles, promoting improved mechanical properties. The treatment is more efficient on samples cured at ambient temperature 7 days, instead of 28 (Figure 3c,d). One possible explanation to this result is that at 7 days the presence of still unreacted material may ease the microstructural transformations induced by the thermal treatment.

The considerations about the effect of biomass are almost the same already made for non-annealed samples: microalgae do not influence deeply the compressive strength, and they seem to be even beneficial in some cases; conversely, the effect of lignin, especially at 7 days, is detrimental.

Therefore, microalgae can be used as filler in geopolymer pastes for 3D printing, without negatively affecting mechanical properties.

### 3.3. Microstructural Analyses

**Figure 4:**
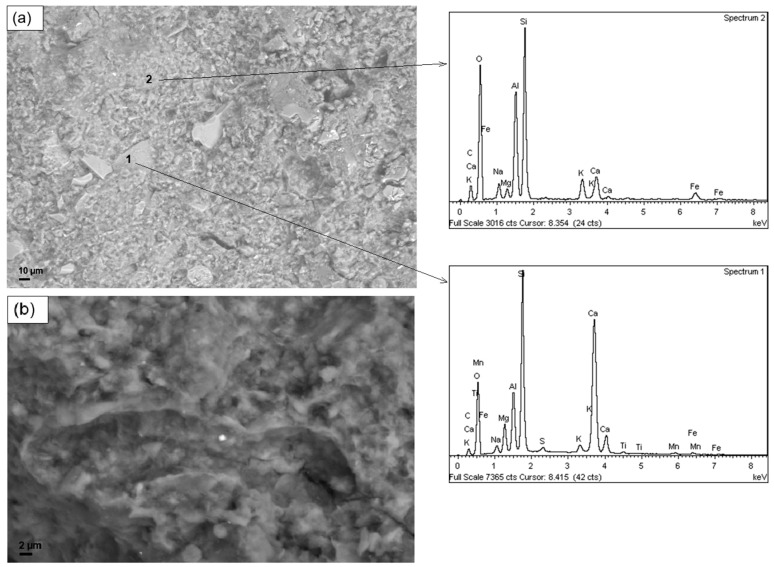
SEM micrographs of the reference sample G-0, at different magnifications: (**a**) SEM micrograph at magnification 1000× and EDS analyses on two spots (number 1 on an inclusion, number 2 on the matrix); (**b**) SEM micrograph at 5000× magnification.

Figure 4 reports the SEM micrographs, at different magnifications, of the sample G-0. In the pictures, inclusions are visible, having irregular shape surrounded by a more homogeneous matrix. Compositional analyses made by EDS showed that the matrix is mainly constituted by silicon, oxygen and aluminum—which are the constituent of the geopolymer network—while the inclusions are based on calcium that can be traced back to impurities of raw materials.

**Figure 5:**
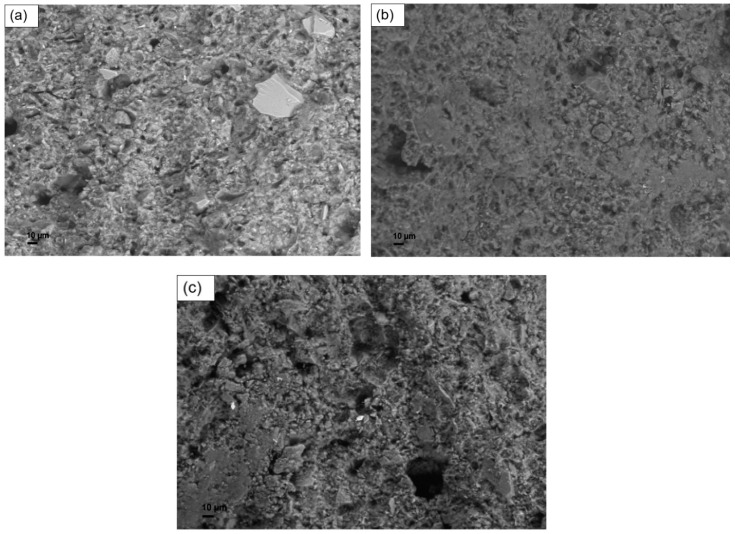
SEM micrographs of samples containing biomass: (**a**) G-S5; (**b**) G-T5; (**c**) G-L5.

SEM micrographs of non-annealed samples containing biomass by 5 php are reported in Figure 5. From the morphological point of view, samples containing microalgal biomass appear similar to the unfilled geopolymer. The similar structure observed, along with the similar composition verified by EDS (see Figure S1 in the Supplementary Materials), is the probable reason of the comparable values of compressive strength recorded between the unfilled material and the formulations containing microalgal biomass.

Conversely, the sample filled with lignin (Figure 5c) shows a less homogeneous microstructure, characterized by clearly visible flocculation and aggregation. The presence of these kinds of defects in the microstructure may be seen as a possible cause of the lower compressive strength of geopolymers containing lignin.

**Figure 6:**
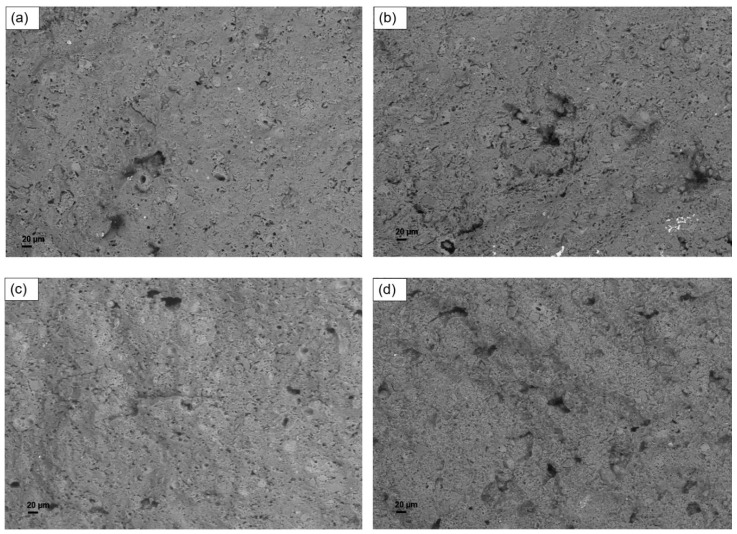
SEM micrographs of annealed samples: (**a**) G-0; (**b**) G-S5; (**c**) G-T5; (**d**) G-L5.

Figure 6 shows the results of SEM analyses performed on the annealed samples. It can be noticed that the microstructure was profoundly modified by the thermal treatment: smoother surfaces are observed, which do not present flocs or agglomerates. The enhanced homogeneity of annealed samples can be related to the predicted effects of sintering between particles and promotion of the reaction of unreacted material. These beneficial effects can be related to the improved mechanical properties previously tested.

Considering porosity, Table 5 reports the results of MIP tests performed on geopolymer samples containing biomass, annealed and not. The classification of pore size is expressed considering pore radii, according to IUPAC indications [40].

Regarding the samples cured at ambient temperature, the unfilled geopolymer (G-0) shows a high amount of porosity (68.5%), predominantly characterized by large voids (larger than 5000 nm). Adding biomass, the total pore volume is reduced to values around 46–48%, regardless the biomass species. In fact, biomass seems able to avoid the formation of air voids, promoting the formation of smaller pores. Specifically, *Spirulina platensis* and *Tetraselmis suecica* have almost the same effect of formation of mesopores (radius between 1.25 nm and 25 nm), while lignin tends to promote macropores (radius between 25 nm and 5000 nm).

MIP analyses on thermally treated samples showed that the total porosity of the unfilled geopolymer is reduced passing from 68.5% to 29.5%, probably because of the already mentioned sintering process that takes place at high temperature. However, the pore size distribution remains very similar, characterized mainly by air voids. Also, in this case, biomass reduces the amount of larger voids, substituted by mesopores and macropores, even if it is no more effective in decreasing the total porosity.

The effect of biomass on porosity can be interesting for applications in which a controlled pore size distribution is required, regarding for example the production of lighter structures, inorganic foams for water filtration or components for thermal insulation.

### 3.4. Large-Scale Printing Test

The formulation with *Spirulina* addition by 5 php was selected for the larger scale printing test, since it guaranteed mechanical properties comparable to the reference geopolymer formulation, it reduced yield stress improving printability and, at the same time, it maximized the amount of end-of-life material used.

Some modifications on the mixture proportions were needed to adapt to a different extrusion mechanism. In particular, to make the material flow more easily, a further reduction of yield stress was required. In order to obtain this result, bentonite amount was reduced (refer to Section 2.2 for the details). Consequently, water amount was decreased, as well.

**Figure 7:**
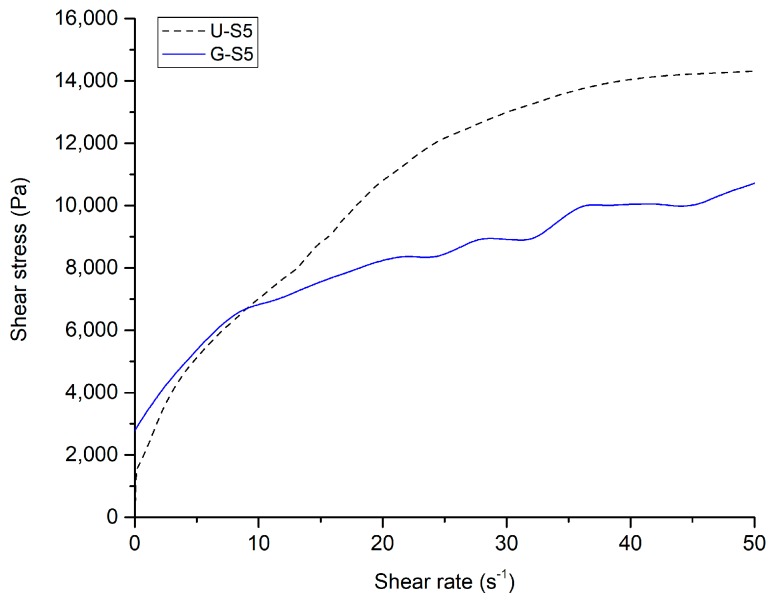
Comparison between the flow curves of the formulations G-S5 and U-S5.

Rheology tests performed on this formulation (U-S5) confirmed the reduction of yield stress with respect to G-S5 (Figure 7). Fitting with Herschel–Bulkley’s model quantified yield stress at 595 ± 72 Pa (R^2^ = 0.997), which is indeed much lower than that of G-S5 (1022 ± 85).

The apparent viscosity value (i.e., the ratio between shear stress and shear rate at each point), for shear rates higher than 10 s^−1^, is higher for the formulation U-S5. This issue, caused by the increase of the viscosity of the dispersing medium due to the reduction of water content, was not a problem during extrusion, since the extruder geometry was optimized for highly viscous fluids.

**Figure 8:**
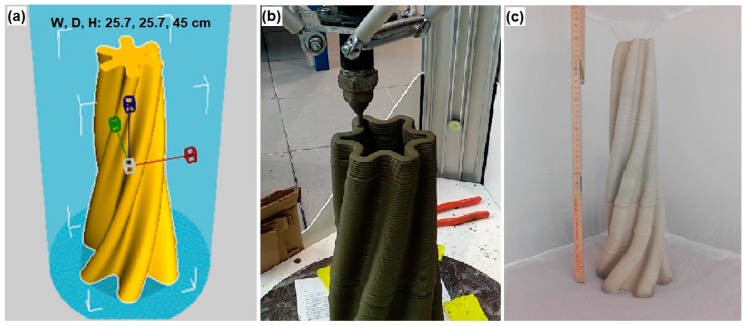
Scale-up process. (**a**) 3D model of the printed object (“W” stands for width, “D” depth, “H” height); (**b**) printing process; (**c**) final result, after 28 days of curing at ambient temperature.

A hollow and self-standing, noncontinuous section structure, having a geometry very difficult to obtain with other manufacturing technologies was selected as demonstrator for the printing test. The 3D model is shown along with the printing process and the final result in Figure 8.

Looking at the final result, the object was successfully printed and it hardened without noticeable problems of shrinkage and cracking.

## 4. Conclusions

This paper discussed the effect of microalgal biomass on the rheological, mechanical and microstructural properties of geopolymers for the application of LDM. Various formulations containing microalgae were tested and the properties were compared with the reference geopolymer mortar and with formulations containing lignin, a different biomass which was taken as a comparison. The main results were the following:

1. Regarding rheology, the yield stress of geopolymer fresh pastes is reduced by the addition of biomass. This effect leads to a general improvement of printability in spite of buildability, which, anyway, remains acceptable in the case of microalgal biomass addition. Therefore, microalgae act as rheology modifiers for LDM-optimized geopolymer concrete.
2. Mechanical properties are only marginally affected by the addition of microalgae up to 5 php. Hence, microalgal biomasses can be used as biofiller in these construction materials.
3. Microstructure is modified by biomass addition, which affects mainly porosity. The total porosity is reduced from 68.5% to values around 46–48% by biomass addition in samples cured at ambient temperature. Moreover, biomass decreases the amount of air pores (i.e., pores larger than 5000 nm), promoting smaller pore conformations in geopolymer materials, such as macropores and mesopores.
4. Annealing, performed at 800 °C for 4 h, improved mechanical properties, by promoting sintering and the reaction of unreacted material.
5. The feasibility of obtaining large size objects was demonstrated by the successful 3D printing of a 45-cm-tall structure.

For the first time in literature, this study demonstrated the possibility of incorporating microalgal biomass wastes into metakaolin-based geopolymer cement paste for additive manufacturing applications, paving the way for a more sustainable disposal of these end-of-life materials.

Further developments of this work are needed to study other important aspects, such as setting time of the cementitious pastes and the long-term durability and weathering resistance of the hardened material, which are of paramount importance when dealing with cementitious materials.

## Figures and Tables

**Table 1:** Chemical composition of Metakaolin Mefisto L05.

Al_2_O_3_ (wt %)	SiO_2_ (wt %)	K_2_O (wt %)	Fe_2_O_3_ (wt %)	TiO_2_ (wt %)	MgO (wt %)	CaO (wt %)
41.1	54.1	0.8	1.1	1.8	0.18	0.13

**Table 2:** Chemical composition of microalgal biomasses used in this work (*Spirulina platensis* and *Tetraselmis suecica*).

Component	*Spirulina platensis*	*Tetraselmis suecica*
Dry Matter ^1^	97.49	95.16
Ash ^2^	9.12	17.05
Proteins ^2^	67.78	37.89
Total sugars ^2^	9.31	14.49
Total free sugars ^2^	2.79	4.19
Lipids ^2^	6.26	12.82
Other ^2^	4.74	13.56

^1^ Dry matter wt % is computed over the total weight of the sample analyzed. ^2^ The component wt % is computed over the weight of the dry matter.

**Table 3:** Mixture proportions of cementitious geopolymer pastes for LDM applications.

Formulation ID	Metakaolin (wt %)	Alkaline Activator (wt %)	Bentonite (wt %)	Water (wt %)	*Spirulina platensis* (wt %)	*Tetraselmis suecica* (wt %)	Lignin (wt %)
G-0	37.6	30.0	22.6	9.8	-	-	-
G-S1	37.0	29.5	22.2	10.6	0.6	-	-
G-S3	36.2	28.9	21.8	11.4	1.7	-	-
G-S5	35.5	28.3	21.3	12.1	2.8	-	-
G-T1	37.0	29.5	22.2	10.6	-	0.6	-
G-T3	36.2	28.9	21.8	11.4	-	1.7	-
G-T5	35.5	28.3	21.3	12.1	-	2.8	-
G-L1	37.0	29.5	22.2	10.6	-	-	0.6
G-L3	36.2	28.9	21.8	11.4	-	-	1.7
G-L5	35.5	28.3	21.3	12.1	-	-	2.8
U-S5	47.7	38.0	10.4	0.7	3.1	-	-

**Table 4:** Herschel–Bulkley’s parameters obtained from the fitting of flow curves.

Sample	Yield Stress (Pa)	K (Pa∙s^n^)	n	R^2^
G-0	2984 ± 55	1717 ± 42	0.35	0.993
G-S1	1195 ± 29	2479 ± 40	0.25	0.952
G-S3	1040 ± 75	2847 ± 56	0.32	0.996
G-S5	1022 ± 85	2605 ± 63	0.34	0.994
G-T1	1737 ± 98	2658 ± 78	0.32	0.992
G-T3	1424 ± 72	3034 ± 54	0.33	0.997
G-T5	2670 ± 123	1733 ± 100	0.42	0.984
G-L5	296 ± 49	1578 ± 37	0.31	0.994

**Table 5:** Results of MIP analyses on geopolymers containing biomass, in terms of total porosity and pore size distribution.

Annealing	Sample	Total Porosity (%) ^1^	Mesopores (r = 1.25 ÷ 25 nm) (%) ^2^	Macropores (r = 25 ÷ 5000 nm) (%) ^2^	Air Voids (r = 5000 ÷ 50,000 nm) (%) ^2^
No	G-0	68.5	2.0	0.4	97.6
	G-S5	47.6	59.5	1.1	39.4
	G-T5	46.0	59.0	1.2	40.1
	G-L5	45.9	4.9	80.8	14.3
Yes	G-0	29.5	0.0	3.3	96.7
	G-S5	45.8	4.4	50.0	45.6
	G-T5	37.1	13.8	34.8	51.4
	G-L5	51.3	4.7	36.1	59.2

^1^ Total porosity is indicated as % with respect to the whole volume. ^2^ The values are reported as % with respect to the pore volume.

## Supplementary Materials

The following are available online at https://www.mdpi.com/1996-1944/12/7/1004/s1, Figure S1: EDS analyses performed on samples containing biomass: (a) G-S5; (b) G-T5; (c) G-L5.
