# Supplementary material for: Additive Manufacturing of Geopolymers Modified with Microalgal Biomass Biofiller from Wastewater Treatment Plants
Source: Materials (Basel). 2019 Mar 27;12(7):1004. doi: 10.3390/ma12071004 (PMC6479326; doi:10.3390/ma12071004)
Supplement: Supplementary file 1 [file materials-12-01004-s001.pdf]

materials

MDPI

Article

Supplementary Materials

Additive Manufacturing of Geopolymers Modified with Microalgal Biomass Biofiller from Wastewater Treatment Plants

Emanuele Agnoli [1,*], Riccardo Ciapponi [2], Marinella Levi [1] and Stefano Turri [1,2]

[1] Department of Chemistry, Materials and Chemical Engineering "Giulio Natta", Politecnico di Milano, Piazza Leonardo da Vinci 32, Milano 20133, Italy; emanuele.agnoli@mail.polimi.it (E.A.); marinella.levi@polimi.it (M.L.); stefano.turri@polimi.it (S.T.)

[2] INSTM – National Interuniversity Consortium of Materials Science and Technology, Via G. Giusti 9, Firenze 50121, Italy; riccardo.ciapponi@polimi.it

* Correspondence: emanuele.agnoli@mail.polimi.it; Tel.: +39 0223994703

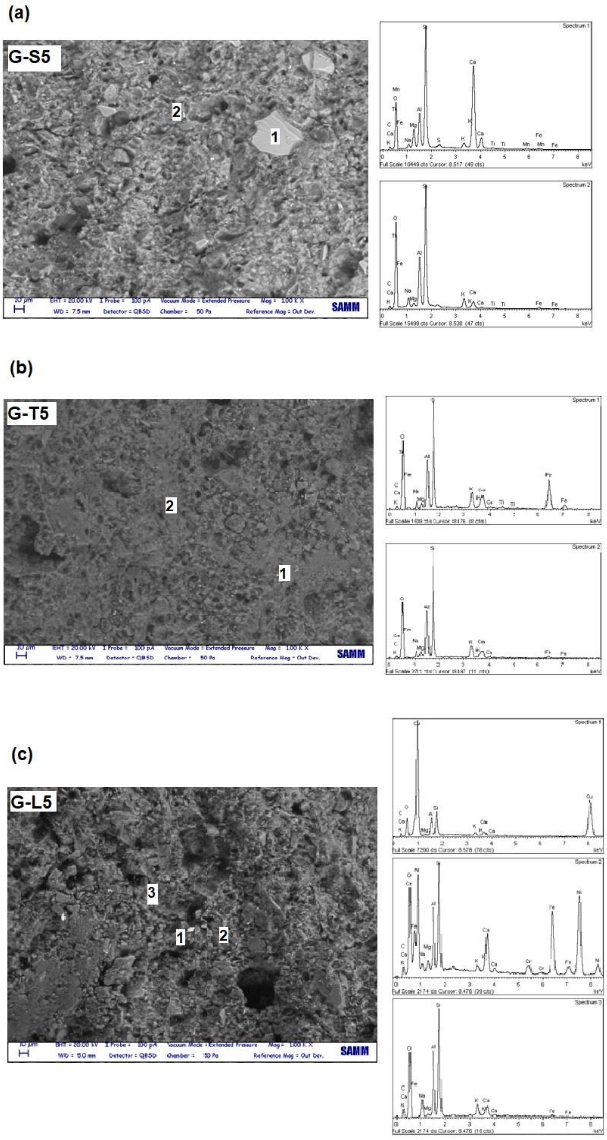

Figure S1. EDS analyses performed on samples containing biomass: (a) G-S5; (b) G-T5; (c) G-L5.
